# Determination of Major and Trace Metals in Date Palm Fruit (*Phoenix dactylifera*) Samples Using Flame Atomic Absorption Spectrometry and Assessment of the Associated Public Health Risks

**DOI:** 10.1155/2024/9914300

**Published:** 2024-01-23

**Authors:** Feven Tamirat, Wondimeneh Dubale Adane, Merid Tessema, Endale Tesfaye, Gizaw Tesfaye

**Affiliations:** ^1^Department of Chemistry, College of Natural and Computational Sciences, Addis Ababa University, P.O. Box 1176, Addis Ababa, Ethiopia; ^2^Department of Chemistry, College of Natural and Computational Sciences, Gambella University, P.O. Box 126, Gambella, Ethiopia; ^3^Department of Chemistry, Fitche College of Teacher Education, P.O. Box 260, Fitche, Ethiopia

## Abstract

This study aimed to assess the concentrations of major and trace metals (Na, Ca, Fe, Zn, Ni, Mn, Cu, Cd, and Pb) in date palm fruit samples collected from diverse regions, including Afar (Ethiopia), Iraq, and Saudi Arabia, utilizing flame atomic absorption spectrometry (FAAS). The wet acid digestion method was employed for sample treatment, with optimization of the key parameters such as reagent volume ratio, oven temperature, and digestion time for analytical applications. Under the optimized parameters, average metal concentrations in date palm fruit samples ranged from 205–299, 134–320, 38.8–115, 25.1–42.2, 9.27–27.9, 7.11–16.3, and 0.002–1.15 mg/kg for Ca, Na, Fe, Ni, Zn, Mn, and Cu, respectively. Cd and Pb levels were below detection limits within the linear range. Generally, date palm samples exhibited higher Ca and Na contents and lower concentrations of Cu and Mn than other metals. Pearson correlation analysis revealed very strong positive correlations between Fe and Na, Na and Zn, Na and Mn, Ca and Zn, Fe and Ni, Fe and Mn, and Mn and Ni. Strong negative correlations were observed for Ni and Na, Fe and Cu, and Cu and Ni. Weak correlations were noted among Na and Cu, Ca and Fe, Ca and Ni, Ca and Mn, Ca and Cu, Fe and Zn, Ni and Zn, Zn and Mn, and Zn and Cu. A recovery study using the spiking method demonstrated acceptable percentage recoveries ranging from 91.6% to 97.8%. Health risk assessment, including chronic daily intake (CDI), hazard quotient (HQ), total exposure hazard index (HI), and carcinogenic risk (CR), indicated CDI, HQ, and HI values below 1.0, except for the HI value for Ni. This suggests that the metals pose no probable public health risk, with the absence of Cd and Pb in date palm samples affirming no carcinogenic threats associated with their consumption.

## 1. Introduction

The date palm (*Phoenix dactylifera*) tree belongs to the family *Arecaceae* and is considered a symbol of life in the desert, as it tolerates high temperatures, water stress, and salinity better than many other trees [1]. It is one of the oldest cultivated trees in arid and semiarid regions. The date palm fruit has tremendous nutritional value due to its rich content of essential nutrients, including minerals, carbohydrates, salts, dietary fibers, vitamins, fatty acids, amino acids, and proteins. It is unique and characterized by certain distinct properties. One of the major features that make the date palm fruit special is that it is the only fruit that can be eaten as a dietary staple in the world and has remained so for thousands of years as a daily diet for millions of people. Another unique property of the date palm fruit is that it can be consumed at each of the three major maturity stages, such as *khalal* (fresh and hard ripe), *rutab* (crisp to the succulent), or *tamar* (fully ripe stage) [2].

**Figure 1:**
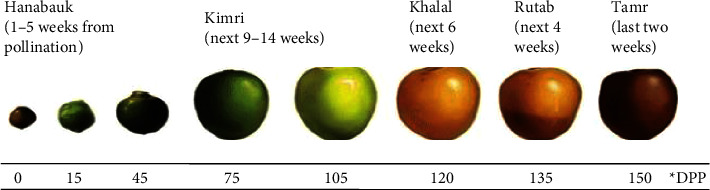
Different fruiting stages of date palm fruit according to days post pollination (DPP), showing *khalal*, *rutab*, and *tamar*, the three edible stages of the fruit [2].

The three major maturity stages are based on the changes in color and nutrient composition of the date palm fruit (Figure 1). To reach the fully ripened, or *tamar,* stage, it takes 150–200 days after pollination. At the *tamar* stage, date fruits have the lowest amount of moisture and are ready for shelf preservation. Fruits are fully ripe, very sweet, dark brown or nearly black, soft, and chewy, with the lowest moisture, and rich in reducing sugars such as fructose, glucose, and sucrose [3, 4].

Date palm fruits have an important role in the diet of people from many countries, and the consumption of date fruits is particularly popular in the Middle East, North Africa, and South Asian countries, where about 90% of the global production of dates takes place [5]. The Food and Agriculture Organization of the United Nations (FAO) has been actively engaged in developing the cultivation of date palms. It recognizes the social, economic, and ecological importance of the date palm in countries, with suitable agroclimatic conditions, where it is traditionally grown [6]. Ethiopia has a high demand for date fruit, mainly during the month of Ramadan and other religious ceremonies and traditional rituals [7]. The date palm was introduced to Ethiopia from Middle Eastern countries about 200 years ago by traders from Yemen and Sudan [8]. From that moment on, it is cultivated mainly by agricultural producers in the Afar, Somali, Gambella, Dire Dawa, and Benishangul-Gumuz regions of the country.

There are more than 5,000 date palm species popular all over the globe [9]. They differ in size, color, texture, antioxidant activity, and phenolic content. Therefore, depending on the nutritional and mechanical characteristics of the fruit, it is possible to use it for various purposes. The date fruit is rich in various types of minerals and health-enhancing nutrients [10]. It contains several vitamins (including B1, B2, and C) with antioxidant, antiviral, and anticancer activities [11, 12]. Moreover, it has substantial amounts of minerals, including Na, K, Mg, Ca, P, Fe, Cu, and Zn. [13].

Minerals can be beneficial to plants at certain levels but can be toxic when levels exceed specific thresholds. Some metals, such as Fe, Zn, Mn, Cu, Co, and Ni, are important micronutrients for plants, but others, including Pb, Hg, Cd, As, Cr, Ga, and Ag, are nonessential for plants and have no known physiological function. The presence of these toxic metals in date fruits above the permissible limit may cause severe health problems for people consuming them [14–16]. Thus, the determination of their levels in date palm fruits is very important for the safety of human health and recommendations for human consumption.

Metal contaminants exist as superficial contaminants on pollutant leaflets of date palm fruit, thereby resulting in a suitable biomonitoring indicator for metal pollution in arid and semiarid areas. The date palm fruits are highly prone to contamination with metals [10]. The increasing metal contamination in fruits is due to the impact of fertilizers, pesticides, and various industrial processes that pollute the water and soil [17]. The ability of plants to accumulate metals in tissues depends on plant size, growth speed, and the productivity of biomass [18, 19].

The consumption of heavy metal-contaminated food items (such as date palm fruits) could pose several health risks to humans, including kidney and liver damage, depletion of immunological defenses and intrauterine growth, psychosocial dysfunctions, anemia, damage to the skin, teeth, and central nervous system, muscular cramps, diseases associated with malnutrition, high blood pressure, and carcinogenic disease. [20–23].

Considering the potential health hazards associated with these metal ions, accurate determination of their concentrations in food samples and comprehensive assessment of the associated public health risks are crucial to safeguard food quality and protect consumer health. The common method for estimating the nature and likelihood of detrimental health effects in individuals exposed to toxic metals is human health risk assessment through chronic daily intake (CDI), hazard quotient (HQ), health risk index (HI), and carcinogenic risk (CR). This approach has been widely used by many researchers to comprehensively estimate the potential hazards due to human health related to exposure to various metals [24–26].

To the best of our knowledge, very few studies have been reported on metal contamination in date palm fruit. Therefore, the aim of this study was (i) to determine the concentrations of major and trace metals (Na, Ca, Fe, Zn, Ni, Mn, Cu, Cd, and Pb) in date palm fruit (*Phoenix dactylifera*) samples collected from three different areas, namely, Afar, Iraq, and Saudi Arabia; (ii) to assess the health risk of trace metals through the determination of chronic daily intake (CDI), hazard quotient (HQ), and hazard index (HI); and (iii) to compare the results of the present study with other previously reported studies on the determination of metals in date palm fruit samples. Furthermore, this experiment aimed at assessing the sugar content of date palm fruit samples.

The present study was limited by the relatively small sample size of date palm fruit (*Phoenix dactylifera*) collected from only three geographical locations and the analysis of only nine major and trace metal elements.

## 2. Materials and Methods

### 2.1. Description of the Study Areas

The study areas were selected based on the rank of the world's leading countries growing fresh dates and also on the current availability of the samples in Addis Ababa, Ethiopia. These selected places include the Afar region (Ethiopia), Iraq, and Saudi Arabia, which are favorable for the growth of the date palm fruit. Afar is associated with an ethnic group inhabiting the Horn of Africa. These people primarily live in the Afar region of Ethiopia and in northern Djibouti, as well as the entire southern part of Eritrea [27]. Iraq, officially the Republic of Iraq, is a country in Western Asia, bordered by Turkey to the north, Iran to the east, Syria to the west, and Saudi Arabia to the south. Saudi Arabia, officially the Kingdom of Saudi Arabia, is a country in Western Asia, constituting the bulk of the Arabian Peninsula [28].

### 2.2. Sample Collection and Preparation

The three varieties of date palm fruit samples were purchased from a local market in Addis Ababa, Ethiopia. The date palm fruit samples were stored in polyethylene bags to keep them free of contaminants. 5 kg of each date palm fruit was cut and deseeded, and the pulp (flesh) portion was carefully washed using tap water and then rinsed using distilled water. The samples were then open-air dried in the laboratory for two weeks to remove as much moisture from the date fruit pulp as possible to inhibit the growth of bacteria, mold, and yeast.

Then, the samples were initially oven-dried at 105°C for 24 hrs to remove the total moisture in the date fruit pulp. The dried date fruit pulp samples were then taken out of the oven and cooled for several minutes. Then, they were ground using a ceramic mortar and pestle to make fine powders, and then the powders were sieved to separate the fine particles of the date fruit pulp from the larger particles. Finally, the finely powdered date fruit pulp samples were then stored in polyethylene bags for further experimental procedures [29].

### 2.3. Chemicals and Reagents

All the chemicals and reagents used in this study were of analytical grade. Metals standard solutions (1000 mg/L), namely, Ca, Na, Fe, Ni, Zn, Mn, Cu, Cd, and Pb, were purchased from Sigma-Aldrich (USA). Intermediate and working standards were prepared from these standard stock solutions. HNO_3_ and HClO_4_ were used for the digestion of the date palm fruit samples. All glassware was properly washed and cleaned, and the reagents used were of analytical grade. Distilled water was used throughout the study.

### 2.4. Sugar Content Determination of Date Palm Fruit Samples

Refractometers measure the degree to which the light changes direction, called the angle of refraction. A refractometer takes the refraction angles and correlates them to refractive index values that have been established. By using this value, one can determine the concentrations of solutions. In the present study, the date fruit pulp pieces (33 g) were placed in 100 mL of distilled water in a glass beaker and heated by using a hotplate (IKA, China) at 85°C for 90 min. The fruits were then separated from the solution by using a metal sieve, followed by squeezing the paste and filtering the solution through fine filters [30]. The solution was passed through a fine metal filter to remove the large fruit tissues. Samples were taken from the solution and cooled for 15 min, and the sugar content was determined by using a J57 automatic refractometer [31].

### 2.5. Optimization of Digestion Procedures

For the digestion of the date palm fruit, different digestion procedures were tested by using a mixture of HNO_3_ and HClO_4_ and varying the volume of the acids, digestion time, and digestion temperature. An optimized procedure was selected to find the values of these parameters that yield the best performance by using a minimum reagent volume and shorter digestion time that gives a clear and colorless solution at a lower temperature for the digestion of the date palm fruit. In this study, the optimization parameters/conditions for the digestion of the three date palm fruit samples are shown in Tables 1–3. As shown in the tables, for the digestion of the date palm fruit samples, 3 mL of HNO_3_ and 1 mL of HClO_4_ (3 : 1 volume ratio), 300°C, and 3 hrs were chosen as the optimum reagent volume, digestion temperature, and digestion time, respectively [29].

### 2.6. Digestion of the Date Palm Fruit

For the determination of metal contents in the date palm fruit, the wet digestion method was performed by using the Kjeldahl apparatus (Gallenkamp, England). 0.5 g of powdered and homogenized date palm fruit samples were weighed and transferred to a 250 mL round-bottomed flask. Based on the optimized parameters, a mixture of 3 mL of HNO_3_ and 1 mL of HClO_4_ (a total of 4 mL) was added to it, followed by fitting the round-bottomed flask to the reflux condenser and heating at 300°C for 3 hrs until it gives a clear and colorless solution. The digested solution was then allowed to cool for 10 mins and 5 mins before and after removing the flask from the condenser, respectively. The cooled solution was filtered using Whatman filter paper to remove any suspended matter. Finally, the filtrate was transferred to a 50 mL volumetric flask, and the volume was made up by using double distilled water. The blank solution was also prepared by using similar procedures on a triplicate basis [29].

### 2.7. Calibration of the Instrument

Metal analysis was performed by using atomic absorption spectrometry (AA-7000 Hitachi) with Deuterium Lamp (D2-lamp) background correction and hollow cathode lamps. The air-acetylene flame was used for the determination of all metals. Four different standard concentrations of metal solutions that include 0.25, 0.5, 0.75, and 1 mg/L for Cu, Zn, and Cd; 0.25, 0.5, 1, and 2 mg/L for Ca, Mn, and Fe; and 1, 2, 3, and 4 mg/L for Ni and Pb were prepared for constructing calibration curves. The regression coefficient (*R*^2^) exhibited good linearity with a value of 0.99. After calibration of the instrument, the digested sample solutions were aspirated into the flame atomic absorption spectrometer, and metal concentrations were determined. Three replicate determinations were carried out for each sample. The same analytical procedure was employed for the determination of metals in the blank samples. The parameters for the calibration of the instrument are shown in Table 4.

### 2.8. Method Detection and Quantification Limits

The method detection limit is the smallest concentration of analyte that can be detected with statistical confidence. The International Union of Pure and Applied Chemistry (IUPAC) defines the detection limit as the smallest concentration of analyte that has a signal significantly larger than the signal arising from a reagent blank. The limit of quantification is the smallest concentration of an analyte that can be reliably determined. The limit of detection (LOD) is expressed as three times the standard deviation of absorbance values (*n* ≥ 10) obtained from the blank divided by the slope of the calibration curve (LOD = 3*∗S*_*b*_/*m*), whereas the limit of quantification (LOQ) is calculated as ten times the standard deviation of absorbance values (*n* ≥ 10) divided by the slope of the calibration curve (LOQ = 10*∗S*_*b*_/*m*) [32, 33], where *S*_*b*_ is the standard deviation of absorbance values obtained from the blank, and *m* is the slope of the calibration curve. In the present study, the detection and quantification limits of the instrument for some metals are given in Table S1.

### 2.9. Trueness of the Method

Several parameters are taken into account to assess the trueness of the method, including linear dynamic range, LOQ, LOD, reproducibility, repeatability, and measurement uncertainty. Recovery is also one of the most commonly used analytical methods for the validation of results and evaluating whether the analytical method is acceptable for its intended purpose. In this study, the FAAS method validation for analysis was performed by using LOD, LOQ, and trueness tests. The trueness of the proposed method was performed by spiking standard solutions of known concentrations of the elements into the samples. The trueness test for each sample was performed in triplicate.

### 2.10. Human Health Risk Assessment

Assessment of human health risks due to the transfer of pollutants to the human body is often performed by using many indices, including chronic daily intake (CDI), hazard quotient (HQ), total exposure hazard index (HI), and carcinogenic risk (CR) [34, 35].

#### 2.10.1. Chronic Daily Intake (CDI)

The daily intake of metals is important to assure the health risks related to the intake of heavy [36] metals from water, food, and exposure to soil. It is expressed in mg/kg/day. The human noncarcinogenic risk effects for each metal can be calculated by using the following equation:

(1) $$\begin{array}{c} \text{CDI }\left(\frac{\text{mg}/\text{kg}}{\text{day}}\right)=\frac{\left(\text{IR}\times \text{CF}\times \text{EF}\times \text{ED}\right)}{\left(\text{BW}\times \text{AT}\right)}, \end{array}$$

where IR is the average daily intake rate of the date palm fruit (kg/person/day), which is 100 g/d (0.1 kg/d) [37, 38], CF is the average concentration of heavy metals in the sample (mg/kg), ED is the exposure duration (the mean life expectancy of a person is 67 years), EF is the exposure frequency (365 days per year), BW is the average body weight (70 kg taken for adults), and AT is the mean exposure period for noncarcinogens (365 days per year × exposure number per year) [39].

#### 2.10.2. Hazard Quotient (HQ)

The hazard quotient (HQ) is the ratio of the exposure of a contaminant to the reference oral dose (RfD) of that contaminant (equation (2), [40]). It indicates noncarcinogenic risk over a lifetime and is used to characterize the risk to human health posed by the intake of metal-contaminated food. If HQ < 1, it is considered that the metal has no potential to cause disease. However, if HQ ≥ 1, it indicates that potential health risks can be expected due to exposure to any specific contaminant [41].

(2) $$\begin{array}{c} \text{HQ}=\frac{\text{CDI}}{\text{RfD}}, \end{array}$$

where CDI represents the chronic daily intake of the sample per day (mg/kg/day) and RfD expresses the reference oral dose of the metal of interest (mg/kg/day). The reference oral dose (RfD) of the metal represents the tolerable daily exposure to any specific contaminant without any major risk of health effects throughout the lifetime of a person [42]. As given by the WHO/FAO (2013) [43], the following RfD values (mg/kg/day) are used in the present assessment: Zn (0.30), Cu (0.04), Ni (0.02), Mn (0.14), Fe (0.70), Pb (0.004), and Cd (0.0005).

#### 2.10.3. Hazard Index (HI)

It is used to evaluate the potential health risks when a person is exposed to more than one heavy metal. The Environmental Protection Agency has developed a hazard index (HI) for the health risk assessment of chemical mixtures [44]. HI can be used as an effective tool to determine the potential health risks that are associated with human exposure to multiple contaminants simultaneously. The HI is the sum of the hazard quotients (HQ) for each contaminant (equation (3), [42]). If the value of HI is greater than 1.0, there would be concern for potential health effects, and if it is below 1.0, it shows no potential health risks of the sample and that the consumers are safe.

(3) $$\begin{array}{c} \text{HI}={\text{HQ}}_{1}+{\text{HQ}}_{2}+{\text{HQ}}_{3}+---\;{\text{HQ}}_{n}. \end{array}$$

#### 2.10.4. Carcinogenic Risk (CR)

The carcinogenic risk (Cr) for any specific contaminant can be calculated by using the following equation. According to USEPA [45], 10^−6^–10^−4^ is the range of permitted lifetime risks for carcinogens.

(4) $$\begin{array}{c} \text{CR}=\text{CDI}\times \text{CSF}, \end{array}$$

where CR represents the cancer risk, CDI is the chronic daily intake calculated in equation (1), and CSF is the cancer slope factor.

## 3. Results and Discussion

### 3.1. Determination of Sugar Content in Date Palm Fruit Samples

The sugar contents of the date palm fruit samples from the different areas were determined by using refractive index values. The refractive index values of the three date palm fruit samples were measured by using an automatic refractometer. The results for refractive index values of the analyzed date palm fruit samples and reported values are presented in Table 5. As shown in the table, the refractive index values of palm fruit samples from Saudi Arabia exhibited a higher index value than those obtained from other sample areas. This indicates that the date palm fruit samples from Saudi Arabia have a higher sugar concentration because the refractive index value increases when the solution gets thicker, resulting in a denser medium that causes higher refraction [46].

### 3.2. Metal Contents in Date Palm Fruit Samples

**Figure 2:**
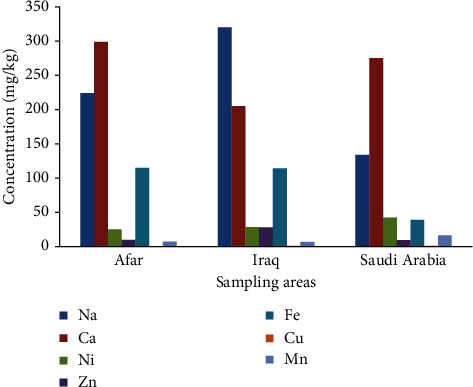
Contents of Na, Ca, Ni, Zn, Fe, Mn, and Cu in the date palm fruit samples collected from different areas of Afar, Iraq, and Saudi Arabia.

The present work presents the major and trace metal concentrations in date palm fruit samples collected from Afar, Iraq, and Saudi Arabia. The determined metals include Ca, Na, Fe, Ni, Zn, Mn, Cu, Cd, and Pb. The mean concentrations of major and trace metals obtained in the studied date palm samples are summarized in Table 6 and Figure 2. The metal contents were determined based on the dry weight of the samples.

As shown in Table 7 and Figure 2, the date palm fruit samples from Afar and Iraq contain a relatively higher concentration of Na than date samples from Saudi Arabia. On the other hand, the date palm fruit samples collected from Saudi Arabia have a higher concentration of Ni than those from Afar and Iraq. The amount of Ca in date palm fruit samples from Iraq was relatively smaller than in other areas. However, a higher Ca concentration was obtained in date palm fruit samples from Afar. Fe was found in higher concentrations in the date palm fruit samples from Afar than the date palm fruits from Iraq and Saudi Arabia. The concentration of Zn in the investigated date samples was in the order of Saudi Arabia > Afar > Iraq. A higher concentration of Mn was observed in the date palm fruit from Saudi Arabia than that from Afar and Iraq. The lowest concentration of Cu in date palm fruit was obtained from Afar. Generally, the variation in metal concentrations within the study area can be attributed to factors such as geographical location, geological activities, industrial facilities nearby, and other influential variables. These factors might contribute to the observed differences in metal levels, providing a comprehensive understanding of environmental dynamics.

In short, the mean concentration of the major metals in date palm samples from Afar was in the order of Ca > Na > Fe > Ni > Zn > Mn > Cu, while the mean concentrations of the major metals in date palm samples from Afar and Saudi Arabia were in the order of Na > Ca > Fe > Ni > Zn > Mn > Cu, and Ca > Na > Ni > Fe > Mn > Zn > Cu, respectively. Generally, the date palm samples from all areas had higher contents of Ca and Na than other metals. However, all date samples contained lower contents of Cu and Mn. In all cases, the trace metals, Cd and Pb, were not detected in the analyzed date palm fruit samples.

### 3.3. Comparison of Results for Metal Contents in Date Palm Fruit Samples in the Present Study with Other Reported Values

As shown in Table 8, in the present investigation, the concentrations of Na and Ca observed in the date palm fruit samples stand out, surpassing the values reported in prior studies, with the exception of samples obtained from Sudan and Nigeria. Similarly, the concentrations of Zn and Mn in the date palm fruit samples obtained in this study were higher than the reported values, except for the samples from Nigeria and Saudi Arabia. Moreover, the Zn concentrations in the date palm fruit samples from the current study were notably elevated compared to the reported values, except for those obtained from Saudi Arabia. It is noteworthy that the amount of Ni in the data samples from this study was higher than the reported values, whereas the Cu content was observed to be the lowest. It is significant to highlight that no traces of the toxic heavy metals Cd and Pb were detected in this study. This distinguishes the date palm fruit samples obtained in the current study from those of Libya, Saudi Arabia, and Egypt, where the presence of Cd and Pb has been reported. The absence of these toxic elements in the examined date palm fruit samples adds a significant dimension to their overall composition, reinforcing their potential for consumption and further emphasizing their safety profile compared to samples from other geographical regions.

The permissible amounts of minerals consumed by an adult from date palm fruit are shown in Table 7. The contents of Na, Ca, and Zn in this study are below the recommended values. This suggests that the date palm fruits are a good source of major metals but not sufficient, so an additional diet from other sources is necessary.

### 3.4. Statistical Analysis

The concept of ANOVA is to compare different sources of variance and make inferences about their relative sizes. In this study, a one-way ANOVA was used to compare the mean concentrations of the metals in the date palm samples from different areas (Afar, Iraq, and Saudi Arabia). Microsoft Excel was also used for data analysis. The results are presented in Table 9. As shown in the table, from the ANOVA results, the values of F_(calculated)_ are less than those of F_(critical)._ This indicates that no significant difference was obtained among the mean concentrations of the metals (Na, Ca, Fe, Zn, Ni, Mn, and Cu) in the date palm fruit samples from the three different sampling areas.

### 3.5. Pearson Correlation Coefficients of Metals

The Pearson correlation coefficient is a measure of the strength of a linear association between two variables and attempts to draw a line that best fits the data of those variables. The correlation coefficient of experimental analysis indicates how strongly two variables are related to each other. A correlation coefficient of +1.0 indicates a perfect positive correlation, while a correlation coefficient of −1.0 indicates a perfect negative correlation. The correlation values are categorized as no correlation (*r* = 0.00–0.19), low correlation (*r* = 0.20–0.39), medium correlation (*r* = 0.40–0.59), higher correlation (*r* = 0.60–0.79), and highest correlation (*r* = 0.80–1.00) [58].

Table 10 presents the correlation coefficient values of the metals in the date palm samples analyzed in this study. Notably, the highest correlation values, encompassing both positive and negative correlations, were observed between Na and Fe, Na and Zn, Ca and Zn, Fe and Ni, Na and Mn, and Fe and Mn. Additionally, significant correlations were observed between Na and Ca, Na and Ni, Fe and Cu, Cu and Ni, and Mn and Cu. Moderate correlations were observed between Fe and Zn, Zn and Mn, and Ca and Cu, while lower correlations were observed in Ca and Fe, Ni and Zn, Ca and Mn, and Zn and Cu. There were no correlations between Ca, Ni, Na, and Cu.

From an analytical or physicochemical perspective, understanding these correlations provides valuable insights into the interactions and interdependencies between the different metals present in date palm samples. This information is crucial for elucidating the potential sources of contamination, metabolic pathways, or environmental influences that may contribute to the observed metal associations. Furthermore, such correlations can aid researchers and analysts in refining analytical methodologies and experimental designs for future investigations, ultimately enhancing the precision and relevance of metal concentration assessments in date palm fruit.

### 3.6. Recovery Study

A recovery study was conducted by spiking known standard solutions of metals to the prepared date palm fruit sample solutions. The percentage recoveries were calculated by using the equation: % recovery = ((amount detected − original amount in the sample)/amount added) *∗* 100. As shown in Table S2, the percent recoveries for the representative four metals analyzed in the date palm fruit samples were found to be within the range of 91.1% –98.5%. This indicates the suitability of the method for real sample analysis^.^

### 3.7. Public Health Risk Assessment Associated with the Consumption of Date Palm Fruit

In the present study, in order to assess the health risks of the consumption of date palm fruit, the potential health risk index values such as CDI, HQ, and HI of different date palm samples were calculated for adults as given in Table 11. From the table, it can be seen that the HQ values of all metals in the date palm samples are below 1.0, which pose no public health risks. Furthermore, the HI values of the date palm samples are lower than 1.0, except for Ni. This indicates that the concentrations of metals in the date palm samples are within the permissible limit and have no probable public health risks except Ni (Table 11). Since Cd and Pb were not detected in the date palm samples, the carcinogenic risk (CR) was not calculated and studied in the present study.

It should be noted that we determined not only the levels of trace (heavy) metals but also the contents of major metals (such as Ca and Na) in date palm fruit samples in the present study. Thus, the concentrations of Ca and Na in date palm fruit samples were determined as indicated in Section 3.2 and Table 7. However, the potential health risk assessments of these metals were not carried out due to their nontoxic nature and the absence of any known adverse health effects associated with their consumption. Other researchers also performed the potential health risk assessment for only heavy metals, not major metals [20, 24–26, 35, 36, 39, 41, 42].

## 4. Conclusion

The investigation of date palm fruits from three regions (Afar, Iraq, and Saudi Arabia) revealed a rich mineral profile, particularly in essential minerals such as Ca, Na, Fe, Mn, and Cu. Adequate levels of micromineral nutrients, such as Ni and Zn, were also observed. Notably, the toxic heavy metals Pb and Cd were not detected in any of the samples. The analytical method employed demonstrated exceptional accuracy, with recovery rates ranging from 91.6% to 97.8%. By comparing metal concentrations among the regions, it is observed that a consistent pattern emerged, with Ca, Na, and Fe surpassing Ni, Zn, Mn, and Cu in mean concentrations. The one-way ANOVA test confirmed no significant differences in these mean concentrations between the regions. Notably, Saudi Arabian date palm samples exhibited the highest sugar content, followed by Iraqi, and the lowest sugar content was observed in Afar samples. The potential health risk assessment indicated no probable public health concerns arising from the consumption of date palm fruits, except for a slightly elevated HI value associated with Ni. Overall, this study provides valuable insights into the mineral composition and safety of date palm fruits from diverse geographical origins. The study provides valuable insights into metal concentrations in date palm fruits, but its limitations include a small sample size, geographical variations, analytical constraints, excluded metals, lack of consideration for temporal variability, and limited exploration of metal sources. These limitations should be considered in interpreting the findings and guiding future research.

## Figures and Tables

**Table 1:** Optimization of reagent volume for the digestion of 0.5 g of date palm fruit samples at a constant temperature and digestion time.

Reagent volume ratio (HNO_3_ : HClO_4_) (total volume = 4 mL)	Temperature (°C)	Digestion time (hrs)	Results (solution color)
2 : 1	300	3 : 00	Colorless and turbid
3 : 1	300	3 : 00	Clear and colorless solution
3 : 2	300	3 : 00	Colorless with suspension
4 : 2	300	3 : 00	Colorless with suspension
5 : 1	300	3 : 00	Slightly yellow
6 : 2	300	3 : 00	Colorless with suspension

**Table 2:** Optimization of temperature for the digestion of 0.5 g of date palm fruit samples at a constant reagent volume and digestion time.

Reagent volume ratio (HNO_3_ : HClO_4_)	Temperature (°C)	Digestion time (hrs)	Results (solution color)
3 : 1	150	3 : 00	Colorless with suspension
3 : 1	180	3 : 00	Colorless with suspension
3 : 1	210	3 : 00	Colorless and turbid
3 : 1	240	3 : 00	Colorless with suspension
3 : 1	270	3 : 00	Colorless and turbid
3 : 1	300	3 : 00	Clear and colorless

**Table 3:** Optimization of time for the digestion of 0.5 g of the date palm fruit samples at a constant volume and digestion temperature.

Reagent volume ratio (HNO_3_ : HClO_4_)	Temperature (°C)	Digestion time (hrs)	Results (solution color)
3 : 1	300	0.50	Light yellow
3 : 1	300	1 : 30	Slightly yellow
3 : 1	300	2 : 00	Colorless with turbid
3 : 1	300	2 : 30	Slightly yellow
3 : 1	300	3 : 00	Clear and colorless solution

**Table 4:** The parameters for the calibration of the FAAS instrument.

Elements	Wavelength (nm)	Standard concentration (mg/L)	Calibration equation	*R* ^2^2^^
Na	589	1, 2, 4, 8	*y* = 0.0891*x* + 0.0276	0.998
Ca	422.7	0.25, 0.5, 1, 2	*y* = 0.0118*x* − 0.0036	0.993
Mn	279.5	0.25, 0.5, 1, 2	*y* = 0.0217*x* − 0.001	0.999
Fe	248.3	0.25, 0.5, 1, 2	*y* = 0.0122*x* − 0.0029	0.992
Ni	232	1, 2, 3, 4	*y* = 0.0045*x* + 2*E* − 05	0.996
Cu	324.7	0.25, 0.5, 0.75, 1	*y* = 0.0118*x* − 0.0036	0.993
Zn	2139	0.25, 0.5, 0.75, 1	*y* = 0.051*x* + 0.0008	0.996
Pb	283.2	1, 2, 3, 4	*y* = 0.0027*x* + 0.001	0.998
Cd	213.9	0.25, 0.5, 0.75, 1	*y* = 0.0273*x* − 0.0016	0.980

**Table 5:** The degree Brix and the refractive index values of the three different sample sites.

Sample sites	Degree Brix (°Brix) (w/w)	Refractive index measured (present work)	Refractive index reported [46]
Afar	14.8	1.3554	1.35536
Iraq	15.2	1.3559	1.35600
Saudi Arabia	15.4	1.3563	1.35632

**Table 6:** The metals contents (mg/kg, *N* = 3) in date palm fruit samples collected from different areas.

Elements	Sample areas					
	Afar		Iraq		Saudi Arabia	
	Concentration (mean ± SD)	%RSD	Concentration (mean ± SD)	%RSD	Concentration (mean ± SD)	%RSD
Na	224 ± 6.5	2.9	320 ± 28.1	8.8	134 ± 9.8	7.3
Ca	299 ± 8.35	2.7	205 ± 7.2	3.5	275 ± 6.2	2.25
Ni	25.1 ± 1.59	6.3	28.2 ± 1.94	6.8	42.2 ± 3.87	9.1
Zn	9.58 ± 0.92	9.6	27.9 ± 2.23	7.9	9.27 ± 0.76	8.1
Fe	115 ± 8.53	7.4	114 ± 4.83	4.2	38.8 ± 3.35	8.6
Cu	0.002 ± 0.0001	4.8	0.9 ± 0.04	5.4	1.15 ± 0.02	1.7
Mn	7.11 ± 0.4	5.6	6.66 ± 0.2	3.0	16.3 ± 0.84	5.1
Pb	ND	ND	ND	ND	ND	ND
Cd	ND	ND	ND	ND	ND	ND

ND, not detected.

**Table 7:** Metal concentrations in date palm fruit (from the present study) and recommended dietary allowances/adequate intakes per adult per day (RDA/AI) values of metals [57].

Metals	Mean concentrations of metals (mg/kg)	Amount of metal a person can get from 100 g of date palm fruit (mg)	RDA/AI values per adult per day (mg)
Na	134–320	13–32	1500
Ca	205–299	21–30	1000
Fe	38.8–115	4.0–12	8.0
Zn	9.27–27.9	0.93–2.8	11

**Table 8:** Comparison of the mean concentration of metals in date palm fruit samples with literature-reported values.

Country	Metal concentration (mg/kg)					Method	Reference
	Na	Ca	Zn	Fe			
Ethiopia (Afar), Iraq, and Saudi Arabia	134–320	205–299	9.27–27.9	38.8–115		FAAS	This study
Nigeria	912–914	372−371	16.0–19.0	11.50–61.5		FAAS	[47]
Malaysia	123–248	488–707	3.28–3.41	8.08–13.5		ICP-OES	[48]
Saudi Arabia	334–338	143–157	0.34–0.45	80.8–203		FAAS/ICP-MAES	[49]
Sudan	567–1318	222–2381	7.50–10.0	6.91–69.1		FAAS	[50]
Pakistan	83.5–103	75.4–93.4	—	0.8–0.82		FAAS	[51]
Libya	19.1–69.0	24.0–64.0	7.40–13.6	8.40–128		FAAS	[52]
Saudi Arabia	—	—	22.8–45.9	56.9–155		ICP-AES	[53]
Pakistan	—	—	—	0.84–1.9		AAS	[54]
Saudi Arabia	73.8–157	150–339	19.5–21.4	21.2–54.9		ICP-OES	[55]

Country	Metal concentration (mg/kg)					Method	Reference
	Ni	Cd	Cu	Mn	Pb		

Ethiopia (Afar), Iraq, and Saudi Arabia	25.1–42.2	ND	0.002–1.15	6.66–16.3	ND	FAAS	This study
Nigeria	—	—	9.84–10.2	10.0–21.0	—	FAAS	[47]
Malaysia	0.149–0.194	—	4.47–5.04	1.62–3.36	—	ICP-OES	[48]
Saudi Arabia	9.60–55.6	0.09–28	1.15–1.26	53.4–53.9	—	FAAS/ICP-AES	[49]
Sudan	—	—	7.1–18.6	5.40–7.8	—	FAAS	[50]
Pakistan	—	—	—	—	—	FAAS	[51]
Libya	—	0.14–1.02	0.54–5.5	—	ND‒0.94	FAAS	[52]
Saudi Arabia	0.82–2.2	0.15–0.26	22.8–52.2	—	0.002–0.006	ICP-AES	[53]
Pakistan	—	0.01–0.02	0.1–0.26	0.09–0.14	0.21–0.29	AAS	[54]
Saudi Arabia	—	—	6.4–7.7	5.8–8.2	—	ICP-OES	[55]
Egypt	0.93–3.0	ND‒0.26	—	—	0.35–2.0	ICP-OES	[56]

ND, not detected.

**Table 9:** One-way analysis of variance (ANOVA) results for the mean concentrations of metals in the date palm fruit samples from the different sampling areas at a 95% confidence level.

Metals	*F*_(calculated)_	*F*_(critical)_	Remark
Na	0.04	5.14	No significant difference among the sample means
Ca	0.03	5.14	No significant difference among the sample means
Fe	0.04	5.14	No significant difference among the sample means
Zn	0.01	5.14	No significant difference among the sample means
Ni	0.07	5.14	No significant difference among the sample means
Mn	0.03	5.14	No significant difference among the sample means
Cu	0.007	5.14	No significant difference among the sample means

**Table 10:** Pearson correlation coefficients (*r*) of metals in date palm fruit samples.

	Na	Ca	Fe	Ni	Zn	Mn	Cu
Na	1						
Ca	‒0.72	1					
Fe	0.85	‒0.26	1				
Ni	‒0.75	0.10	‒0.98	1			
Zn	0.88	‒0.96	0.50	‒0.35	1		
Mn	‒0.87	0.31	‒0.99	0.97	‒0.54	1	
Cu	‒0.18	‒0.53	‒0.67	0.78	0.29	0.63	1

**Table 11:** CDI (mg/kg/day), RfD (mg/kg/day), HQ, and HI for the date palm samples collected from different areas.

Elements	Sampling areas	CDI	RfD	HQ	HI
Ni	Afar	0.0066	0.02	0.33	1.25
	Iraq	0.0074		0.37	
	Saudi Arabia	0.011		0.55	

Zn	Afar	0.0025	0.30	0.0083	0.04
	Iraq	0.0073		0.024	
	Saudi Arabia	0.0024		0.008	

Fe	Afar	0.03	0.7	0.043	0.1
	Iraq	0.03		0.043	
	Saudi Arabia	0.01		0.014	0.0135

Cu	Afar	0.00000052	0.04	0.000013	
	Iraq	0.00024		0.006	
	Saudi Arabia	0.0003		0.0075	0.057

Mn	Afar	0.0019	0.14	0.014	
	Iraq	0.0017		0.012	
	Saudi Arabia	0.0043		0.031	

## Data Availability

The data used to support the findings of the study are included within the article.

## Supplementary Materials

Supplementary Materials Supplementary 1. Table S1: the detection and quantification limits of the instrument for the determination of metals in date palm fruit samples. Supplementary 2. Table S2: recovery test result (*N* = 3) for the determination of four metals in date palm fruit samples using the FAAS method.
