# Supplementary material for: Determination of Major and Trace Metals in Date Palm Fruit (Phoenix dactylifera) Samples Using Flame Atomic Absorption Spectrometry and Assessment of the Associated Public Health Risks
Source: Int J Anal Chem. 2024 Jan 23;2024:9914300. doi: 10.1155/2024/9914300 (PMC11325699; doi:10.1155/2024/9914300)
Supplement: Supplementary Materials — Supplementary 1. Table S1: the detection and quantification limits of the instrument for the determination of metals in date palm fruit samples. Supplementary 2. Table S2: recovery test result (N = 3) for the determination of four metals in date palm fruit samples using the FAAS method. [file 9914300.f1.docx]

**Supplementary Material**

**Determination of Major and Trace Metals in Date Palm Fruit (*Phoenix Dactilifera*)** **Samples Using Flame Atomic Absorption Spectrometry and Assessment of the Associated Public Health Risks**

**Feven Tamirat^1^, Wondimeneh Dubale Adane^1^, Merid Tessema^1^*, Endale Tesfaye^2^, Gizaw Tesfaye^3^,**

^1^Department of Chemistry, College of Natural and Computational Sciences, Addis Ababa University, P.O. Box 1176, Addis Ababa, Ethiopia

^2^Department of Chemistry, College of Natural and Computational Sciences, Gambella University, P.O. Box 126, Gambella, Ethiopia

^3^Department of Chemistry, Fitche College of Teacher Education, P.O. Box 260, Fitche, Ethiopia

*Corresponding author. E-mail: [tessmer2265@yahoo.com](mailto:tessmer2265@yahoo.com)

Table S1: The detection and quantification limits of the instrument for the determination of metals in date palm fruit samples.

| Metal | LOD^*^ (mg/kg) | LOQ^*^ (mg/kg) |
| --- | --- | --- |
| Na | 12 | 40 |
| Ca | 0.55 | 1.82 |
| Ni | 0.83 | 2.74 |
| Zn | 0.36 | 1.20 |
| Fe | 0.21 | 0.70 |
| Mn | 0.44 | 1.47 |
| Cu | 0.13 | 0.43 |
| Pb | 0.05 | 0.17 |
| Cd | 0.07 | 0.23 |

^*^LOD: Limit of detection; ^*^LOQ: limit of quantification

Table S2: Recovery test result (N=3) for the determination of four metals in date palm fruit samples using the FAAS method.

| Elements | Original concentration  in the sample (mg/kg) | Concentration  added (mg/kg) | Concentration  detected (mg/kg) | % Recovery |
| --- | --- | --- | --- | --- |
| Na | 9.58 | 4.79 | 14.3 | 98.5 |
| Ca | 299 | 74.7 | 369 | 93.7 |
| Fe | 115 | 40.3 | 152 | 91.8 |
| Zn | 224 | 56.0 | 275 | 91.1 |
